# Resilience in a Hypoxic World: Fish Respond Through Plasticity in Their Behaviour, Whereas Adaptation and Adaptation of Plasticity in the Behaviour and Metabolism Occur

**DOI:** 10.1002/ece3.73128

**Published:** 2026-02-24

**Authors:** Ludovic Toisoul, Alycia Valvandrin, Luisa Bermejo Albacete, Katja Anttila, Amélie Crespel

**Affiliations:** ^1^ Department of Biology University of Turku Turku Finland

**Keywords:** metabolism, multigenerational hypoxia, recent evolution, risk‐taking, sociability, sticklebacks

## Abstract

The occurrence of climate change‐induced hypoxia, that is, low dissolved oxygen levels in water, is increasing at an unprecedented rate. When organisms cannot escape, they must cope through plasticity, within or across generations, or even locally adapt. Documenting all these responses is essential to better understand the populations' capacity to persist in changing environments over generations. Therefore, two populations of sticklebacks ( *Gasterosteus aculeatus* ), one exposed to frequent hypoxia in the wild and one not, were bred for two generations, exposing offspring to either normoxia or daily fluctuating hypoxia (35% DO at night). When exposed to hypoxia within a generation, fish were less social and took fewer risks. However, fish from the population previously exposed to hypoxia in the wild were, on the contrary, more social and took more risks while also decreasing standard metabolic rate and growth, showing signs of adaptation. Fish also showed adaptation of their plasticity by losing plasticity for their hypoxia tolerance thresholds. No intergenerational plasticity was revealed. Overall, our study revealed that fish were able to cope with hypoxia within and across generations mainly through within‐generation plasticity on behaviour, potentially giving time before adaptation could take place.

## Graphical abstract

Our study investigates the processes at play behind hypoxia response by looking at the independent and combined roles of plasticity and evolution, while also considering inter‐generational plasticity. We used two fish populations exposed to different levels of hypoxia in the wild and experimentally exposed to two levels of hypoxia conditions. Our main findings revealed that fish response to hypoxia might rely on within‐generation plasticity in their behaviour until genetic adaptation occurs. Although signs of adaptation were observed, not only in the fish behaviour and metabolism but also on the capacity for plasticity in their anaerobic metabolism, no strong inter‐generational plasticity seemed to take place.

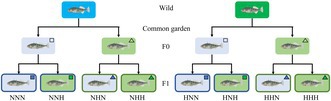

## 1 Background

The increasing occurrence of hypoxic events (i.e., low dissolved oxygen levels in water potentially impacts the behaviour and physiology of aquatic organisms (Pollock et al. 2007)) is one of the biggest climate change threats to freshwater ecosystems. Hypoxia has always occurred naturally, especially during the nights, because of plants and aquatic organisms' oxygen intake (Pollock et al. 2007; Mallin et al. 2006; Flint et al. 2012). However, the combination of recent anthropogenic activities has increased the intensity and frequency of hypoxia events. These activities notably include organic water pollution and eutrophication, increasing algae and aquatic plants production, and global climate change, with its associated increase in water temperature, reducing the oxygen solubility in water (Pollock et al. 2007; Jenny et al. 2016; Sampaio et al. 2021; Deutsch et al. 2015). The increased occurrence of hypoxia is having strong consequences on freshwater organisms and ecosystems (Jenny et al. 2016; Altieri and Gedan 2015). As oxygen availability is one of the most crucial environmental parameters for aerobic organisms, a decrease can affect most of the organisms' physiological functions with an overall impact on their fitness and survival. This can ultimately induce mass mortality events and community extinctions (Pollock et al. 2007). When species are facing challenging environments, their first response is to migrate to a more suitable environment. However, migration is not always possible, especially in enclosed environments such as freshwater ponds, where organisms need to cope with local conditions to avoid extinction.

When directly exposed to hypoxia, organisms can respond to the new environmental condition through phenotypic plasticity (Earhart et al. 2022; McBryan et al. 2013). Phenotypic plasticity is the capacity of an organism to express different phenotypes depending on environmental conditions, using a single genotype (Pfennig et al. 2010). Several traits playing a crucial role for the fish's survival could be affected. For instance, energy production and use of aerobic metabolism could first be reduced, but if this is insufficient to meet energy needs, anaerobic metabolism may be increased to sustain energy production (Borowiec et al. 2018). Indeed, previous studies have shown that fish can decrease their maximal metabolic rate and metabolic scope to conserve energy during hypoxic events (Yang et al. 2013), but the decrease was lower when the fish were previously exposed to fluctuating hypoxia. In addition, their overall hypoxia tolerance and capacity to maintain their aerobic metabolism activity at lower oxygen concentration, the critical oxygen tension (*P*_crit_) was improved compared to fish only exposed to a normoxic environment (Yang et al. 2013; Dan et al. 2014). Such modifications in fish metabolism could have repercussions for the daily activities of the fish that are crucial for survival, such as the fish's growth and swimming capacity, but also diminishing their capacity to take risks in their environment and to interact with conspecifics, which can all be involved in avoiding potential predators (Domenici et al. 2017; Braga et al. 2013). However, so far, little is known about the impact of fluctuating hypoxia on these traits. Although such plasticity offers a quick response to environmental changes within a generation, this can come at a cost for the fitness of the organism and is limited by the possibility of the genome (Murren et al. 2015). Therefore, plasticity alone might not be sufficient for organisms to cope with the upcoming environmental changes that are expected to continue across generations.

When the ongoing hypoxic changes persist, organisms may adapt via phenotypic evolution, leading to changes at the genome level through natural selection. Although evolutionary adaptation leads to more permanent responses to cope with new, changing environments, it requires several generations to occur. Recent studies revealed that different fish species could evolve in response to hypoxia, with freshwater tropical fish showing adaptation in their gill morphology and lower aerobic energy metabolism (Crampton et al. 2025) and fish from high altitude lakes in the Tibetan plateau, showing also adaptation for lower aerobic energy metabolism and increase in body size (Chen et al. 2020; Yang et al. 2021; Zhang et al. 2017). However, although these adaptations were observed in response to an exposition over millions of years, there is still little information if hypoxic environments can lead to adaptation in a faster timeframe such as climate change. In addition, adaptation can also affect the population capacity for phenotypic plasticity. Indeed, plasticity itself can evolve, with selection toward higher plasticity in populations living in more variable environments compared to populations living in stable environments (Murren et al. 2015). Thus, populations that experienced fluctuation in their past environment may have quicker and stronger plastic responses when facing similar environmental variation than populations previously living in more stable environments (Burton et al. 2022; Reed et al. 2011). Therefore, it is important to understand not only how adaptation to past environments shapes the phenotype of future generations, but also their plastic responses.

Going beyond the dichotomy between direct plasticity and evolutionary response, inter‐ and transgenerational plasticity (i.e., the impact of respectively the parental and grand‐parental environment on the offspring's phenotypic expression, transmitted through non‐genetic mechanisms) can also occur (Donelan et al. 2020; Harmon and Pfennig 2021; Bonduriansky et al. 2012; Bautista and Crespel 2021). These non‐genetic inheritance mechanisms can include epigenetic marker inheritance (e.g., DNA methylation, histone modification or small RNAs) as well as parental hormones, oocyte mitochondria and nutrient content (Bonduriansky et al. 2012; Ryu et al. 2018). If the phenotype transmitted through intergenerational effect enhances the organism's fitness, it could help populations to cope with environmental changes across generations (Harmon and Pfennig 2021). In addition, it could facilitate phenotypic evolution by buffering the effects of the environment until genetic assimilation occurs (Harmon and Pfennig 2021; Bateson et al. 2014). So far, it has been observed that a parental generation exposed for a month to mild constant hypoxia can increase hypoxia tolerance of their first offspring generation during the larval stage (Ho and Burggren 2012; Ragsdale et al. 2022). However, there is little information on the potential intergenerational effect of fluctuating hypoxia on the fitness of offspring at later developmental stages. It is only by looking at both early and late developmental stages that the long‐lasting repercussions of the parental condition on their offspring's fitness could be identified.

The goal of this paper was to investigate the potential physiological and behavioural responses of a freshwater fish (three spined sticklebacks, *Gasterosteus aculeatus* ) facing fluctuating hypoxia within and across generations. To do so, we used the offspring of two distinct wild populations originating from a normoxic and a hypoxic freshwater environment. The offspring were themselves reared in a common garden either in normoxic or daily fluctuating hypoxic environments. Using the second generation produced in captivity allowed us to document the effect of the wild population (i.e., adaptation), parental (i.e., intergenerational plasticity), and present (i.e., direct plasticity) environments, as well as their interaction, on the offspring adult phenotype. We measured a range of fitness‐related traits such as their aerobic metabolism (standard metabolic rate and aerobic scope), their recovery post‐exercise (excess post‐exercise oxygen consumption, EPOC), their hypoxia tolerance thresholds (loss of equilibrium and *P*_crit_), as well as their overall growth, swimming performance, and behaviour (risk‐taking and sociability). Our main questions were: (1) How did the present fluctuating hypoxic environment influence the expression of the different fitness‐related traits? (2) Did the wild population environment have an impact on the phenotypic expression, and did it shape the plastic responses of the offspring? (3) What was the impact of the parental environment on the offspring's adult traits expression? We hypothesised that fish reared in present fluctuating hypoxia would display a decrease in their aerobic scope, risk‐taking and social behaviour to conserve energy, as well as a higher hypoxia tolerance with an improved *P*_crit_. We expected to observe signs of adaptation to hypoxia in the fish population exposed to hypoxia in the wild, with fish from this population having a lower standard metabolic rate, but also signs of adaptation of their plasticity, with fish from this population showing a stronger plastic response to our experimental hypoxia. Finally, offspring from parents reared in fluctuating hypoxia were anticipated to have improved hypoxia tolerance thresholds. The combined investigation of plasticity, adaptation, and intergenerational inheritance will give invaluable insights into the integrated responses of freshwater fish to hypoxic events within and across generations.

## 2 Methods

### 2.1 Fish Populations and Rearing Environment

The fish used for the experiment were the second offspring generation of two wild populations of freshwater three‐spined sticklebacks ( *G. aculeatus* ) collected in September 2020 around Hamburg, Germany. About 200 wild fish were collected from Ottersbek stream (53.576° N, 9.965° E) that experienced strong summer hypoxia events (as low as 1.1 mg O_2_ l^−1^) for at least 10 years (Prokkola et al. 2015) (Table S1 and Figure S1), forming the hypoxia population. About 400 fish were collected from the Lottbek river (53.684° N, 10.136° E) that never experienced hypoxia (oxygen level always above 6.7 mg O_2_ l^−1^, Table S1 and Figure S1), forming the normoxia population. The wild fish caught from the two populations were transported and reared at the fish facilities of the Department of Biology of the University of Turku. The fish were housed in six 100 L glass tanks filled with fully oxygenated dechlorinated freshwater, with shelters, and fed daily with bloodworms (red mosquito larvae).

**FIGURE 1:**
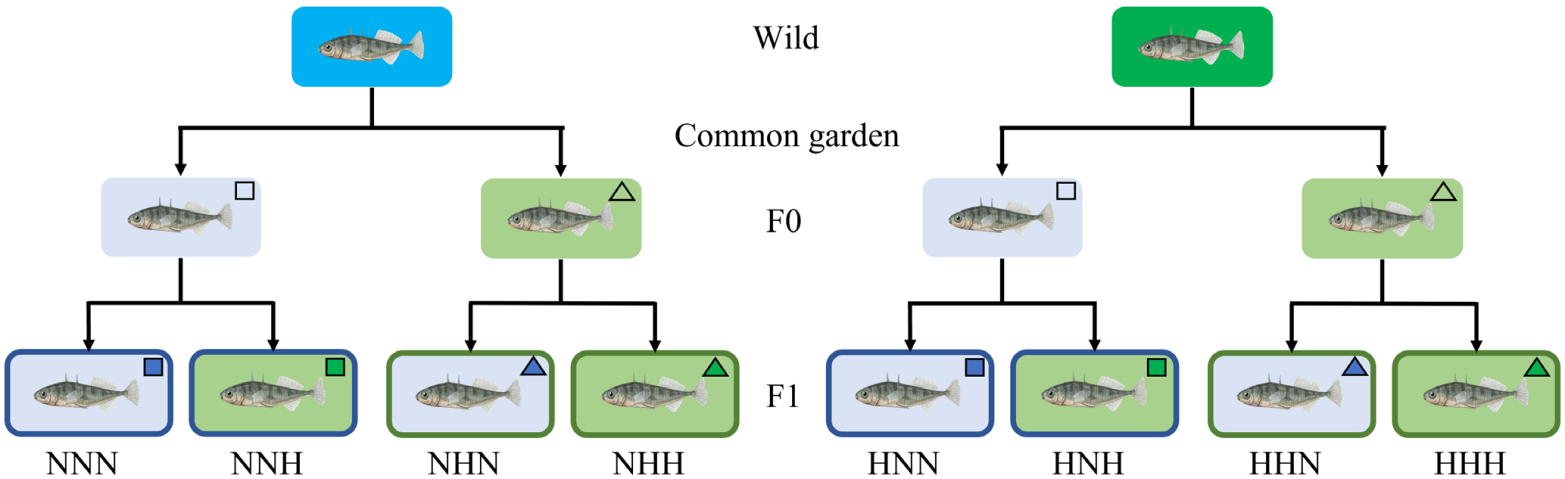
Design of the experiment. Wild fish populations from normoxic environments are in blue background and from hypoxic environments in green. The fish were brought to the University of Turku and reproduced to create the F0 and F1 generations. The rearing environment is shown by the colour of the box background, with a light blue when the fish were reared in a normoxic environment and light green when the fish were reared in a fluctuating hypoxic environment. In addition, for F1, the rearing environment is also indicated by the colour of the shape, with dark blue for normoxic environments and dark green for fluctuating hypoxic environments. Finally, the F0 parental environment is represented by the outline of the box and the form of the shape on the top right corner, a dark blue and square shape for normoxic environments, and dark green and a triangle for fluctuating hypoxic environments. The first letter of the group name, either normoxia (N) or hypoxia (H), represents the two wild populations. The second and third letters represent the parental and present environment, either normoxic (N) or hypoxic (H), respectively.

The wild fish were used to start a multigenerational experiment (Figure 1). In total, 23 and 22 females (from the normoxia and hypoxia population, respectively) were artificially reproduced with 16 and 14 males (from the normoxia and hypoxia population, respectively) between June and July 2021 to create 40 families in the first generation (F0) produced in captivity for each population. Each batch of eggs were divided equally to two environmental conditions: a normoxic environment, with a minimum oxygen level of 90% air saturation (9.9 ± 0.1 mg O_2_ l^−1^) throughout days and nights, or a fluctuating hypoxic environment, with a minimum oxygen level of 90% air saturation during the day (from 7:00 to 19:00) and about 35% (3.7 ± 0.6 mg O_2_ l^−1^) during the night (from 19:00 to 7:00). Therefore, four groups were created in the F0 generation depending on the wild populations (normoxia or hypoxia population) and their rearing environment (normoxic or fluctuating hypoxic environment). Once the fish from the F0 generation were ready to breed, between June and July 2022, 12 females and 6 males from the hypoxia population reared in normoxic environment were used to create 12 families, 11 females and 6 males from the hypoxia population reared in fluctuating hypoxic environment were used to create 11 families, 13 females and 7 males from the normoxia population reared in normoxic environment were used to create 13 families, and finally 15 females and 7 males from the normoxia population reared in fluctuating hypoxic environment were used to create 15 families. In total, 51 families were created in this offspring generation (F1). Again, each batch of eggs from each family was divided equally and exposed to the two environmental conditions: normoxic or fluctuating hypoxic environments, as previously described. Therefore, eight groups were used in the F1 generation, depending on their wild population, parental, and present environment. The normoxic environment was maintained by constant oxygenation of water using air stones. The daily fluctuating hypoxic environment was created by diffusing nitrogen gas in the water using a feedback control system (Loligo Systems, Viborg, Denmark) connected to an oxygen probe. The daily cycles of hypoxia were controlled by a timer. The water oxygen concentration was recorded every 30 s using Firesting O_2_ optical oxygen meters and probe sensors (PyroScience GmbH, Germany).

The eggs and larvae from each family were reared in 1 L mesh boxes placed in two 500 L rearing systems (one for each experimental condition), which were divided into five racks. The systems were supplied with dechlorinated and thermoregulated freshwater maintained at 17°C. The photoperiod was set at 12L:12D. In the first 2 months, the larvae were fed three times a day with dry food (JBL NovoTom Artemia) and once a day with 24 h‐old *Artemia* sp. Once the larvae were 2 months old, they were fed three times a day using dry food (Tetra TetraMin Baby) and once a day with 48 h‐old *Artemia* sp. After 6 months, 10 fish per family per group were randomly taken and individually tagged near the dorsal fin using visible elastomers (VIE, North West Marine Technology, WA, USA). The fish were then transferred into 5 L flow‐through tanks containing shelters, with equal representation of all the families and groups within the tanks in each experimental condition. The fish were fed *ad libitum* twice a day using dry flakes (Tetra TetraMin Flakes). Before each measurement, the fish were fasted for 24 h. The fish were also left undisturbed for at least a week between any measurements. All the measurements took place between January and April 2023 on 30 to 34 fish per group, with equal representation of the families. The exact number of fish used for each group and each phenotypic trait collected is shown in the Table S2. All measurements were made at a constant temperature of 17°C in fully oxygenated water.

### 2.2 Metabolic Rate

Fish individual *M*O_2_ consumption was measured using intermittent flow respirometry (Killen et al. 2021). The setup comprised 16 glass chambers of 61.5 mL immersed in a 40 L tank. Oxygen consumption within each chamber was recorded using a probe sensor, within a probe holder connected to the chamber, and connected to FireSting‐PRO 4 Channels (Pyroscience GmbH, Germany) using Pyro Workbench V1.5.0 software (Pyroscience GmbH, Germany) to monitor the oxygen consumption. The chamber and probe holder were kept in a closed recirculation loop using a peristaltic pump (Masterflex LS model 7534‐08). The probe's 100% calibration was performed every day before each trial. The data were recorded every 4 s. Immersed pumps were used to flush the chambers for 2 min every 10 min with new oxygenated water to create measuring cycles. Open‐close cycles were controlled by AquaResp3 software. Between each trial, the system was cleaned using 200 mL of bleach and rinsed at least three times using clean water to avoid the development of bacteria. The microbial background respiration was monitored before and after each trial in the chambers without the fish for at least three cycles. In addition, one chamber was left empty for each trial to control it.

Prior to the measurement, the fish mass and total length were measured. A maximum of 15 fish were used for each trial, leaving one chamber empty to measure the microbial respiration during each trial. In total, 251 fish were measured over 17 days. At the beginning of the trial, each fish was actively chased to exhaustion in a bucket for 2 min and then rapidly placed inside a chamber to measure its maximal metabolic rate (MMR) post exhaustion. The fish were left in their respective chambers overnight (i.e., about 16 h) to measure their standard metabolic rate (SMR). The setup was covered during the SMR measurement, and the lights were turned off to avoid any disturbances. The following day, the critical oxygen tension (*P*_crit_) was measured. The oxygen was gradually decreased, from 100% to 20% air saturation with intermediate steps at 80%, 60% and 40%, by diffusing nitrogen gas in the water controlled by an oxygen analyser and probe (Loligo Systems, Viborg, Denmark). Two 10‐min cycles were measured for each step. After the last cycle at the 20% air saturation, the chambers were closed and disconnected from the flush cycles, and the fish were left to consume the remaining oxygen inside the chamber down to 4% air saturation. Once a fish lost its equilibrium and became unresponsive to three distinct consecutive stimuli by gently rotating the chamber underwater, it was removed and placed in a recovery tank with oxygenated water. The fish recovery was monitored up to 24 h after the trial and no mortality was reported.

The fish oxygen consumption *M*O_2_ (mg O_2_ h^−1^) was calculated using the declining oxygen slopes for each cycle extracted from raw oxygen measurement using the software LabChart Reader 8.1.27 (ADInstruments, NZ). The slopes were corrected by the microbial background respiration. The fish *M*O_2_ was then calculated using this equation: *M*O_2_ = corrected slopes × (chamber total volume—fish mass (g)). The MMR was calculated by determining the highest *M*O_2_ obtained from the rolling slope method (using 2‐min slopes to calculate *M*O_2_ every 4 s) in the first slope obtained after the chase. Fish SMR was determined as the 0.2 quantile of the *M*O_2_ measurements after the first 5 h of fish measurements inside the chambers. The aerobic scope (AS) was determined as the difference between MMR and SMR. The *P*_crit_ (mg O_2_ l^−1^) of the fish was calculated using a R script from Claireaux and Chabot (2016) that established a linear regression between the water oxygen level and the fish *M*O_2_ during the oxygen reduction. The intersection between the regression line and the fish SMR was determined as the *P*_crit_ value. The excess post‐exercise oxygen consumption (EPOC, mg O_2_ h^−1^) was calculated as the integral of the area between the fish *M*O_2_ and SMR during the recovery period post‐MMR and until the oxygen consumption reached the value of SMR + 10% for two consecutive measurements (Zhang et al. 2018).

### 2.3 Hypoxia Tolerance

The time of loss of equilibrium (LOE) in the hypoxia challenge was assessed using a 50 L tank thermoregulated at 17°C. Each day, three distinct trials of hypoxia challenges were conducted, and the fish were tested in groups of 32, that is, 16 fish from the normoxic and 16 fish from the fluctuating hypoxic environment within the same challenge. The fish were then placed in four 1 L mesh boxes, which were arranged on the top layer of the tank on both sides. Two water pumps were set in each tank to ensure the homogeneity of the water condition within the tanks. The desired oxygen level in the system was reached by controlling the flow of nitrogen gas diffusing into the water using a feedback controller connected to an oxygen analyser and probe (Loligo Systems, Viborg, Denmark), and was additionally monitored using FireSting O_2_ optical oxygen meters and probes (FireSting‐PRO). After that, the fish were placed in the boxes and were left undisturbed for 1 h at an oxygen level of 100% air saturation. The oxygen level was then rapidly decreased from 100% to 21% air saturation over 20 min, followed by a gradual decrease of 2% every 5 min until reaching 9% air saturation, and finally a slight decrease of 1% every 10 min. Once a fish lost its equilibrium, that is, when the fish was no longer able to recover normal positioning and was unresponsive to three consecutive stimulations, it was carefully removed, identified, put in a fully oxygenated recovery tank, and the corresponding time at LOE was recorded. The oxygen level at LOE was then calculated using the equation LOE (% air saturation) = (*O*–(*T*/*T*_*i*_ × *O*_*i*_)), where *O* (% air saturation) is the lowest oxygen decrement completed before the loss of equilibrium, *T* (s) is the time at which the fish loss equilibrium in the last decrement, *T*_*i*_ (s) is the timespan between each decrement (300 s for 20% to 12% and 600 s for 10% to 2%) and *O*_*i*_ (% air saturation) is the oxygen decrease between each decrement (2% from 20% to 12% and 1% decrease between 9% and 2%).

### 2.4 Growth

The specific growth rate (SGR) for the length of the fish was measured over 3 months, from January to March. The total length (to the nearest 0.01 mm) of the fish was used. The SGR (% mm day^−1^) of each fish was then calculated according to SGR = (Ln*L*_*f*_—Ln*L*_*i*_) × 100/*T*, where *L*_*f*_ is the final length, *L*_*i*_ is the initial length, and *T* is measured in days between the two measurements.

### 2.5 Swimming Performance

The critical swimming speed (*U*_crit_) of the fish was assessed using a 90 L Steffensen‐type swimming tunnel (Loligo Systems, Viborg, Denmark) filled with water thermoregulated at 17°C and shielded from surrounding disturbances. Each day, four swimming trials were conducted, and the fish were tested in groups of 16 from either normoxic or fluctuating hypoxic environments. At the start of each trial, the group of fish was placed into the flume and left undisturbed for 20 min at a speed of 3 cm s^−1^. After that, the water velocity increased in increments of 3 cm s^−1^ every 5 min. Once a fish showed signs of fatigue, that is, stopped swimming, stayed in contact with the rear screen of the flume and was unresponsive to three stimulations to help it swim, it was carefully removed from the swimming tunnel through a small opening on the lid at the rear of the flume. The fish were then identified, and the corresponding time of fatigue and water velocity were recorded. The total length of the fish was also taken at the end of the trial. The *U*_crit_ (body length s^−1^) was then calculated using the equation *U*_crit_ = (*U*+(*T*/*T*_*i*_ × *U*_*i*_))/*L*, where *U* (cm s^−1^) is the highest velocity maintained in a complete time interval, *T* (s) is the time between the last completed increment and the fish stopped swimming because of exhaustion, *T*_*i*_ is the time between each increment (300 s), *U*_*i*_ is the speed increment (3 cm s^−1^), and *L* is the length of the fish (mm).

### 2.6 Behavioural Assays

Individual fish behaviours were tested through risk‐taking assays, consisting of shelter and open field assays, and sociability assays. Each fish went through the assays for two distinct rounds with at least 2 weeks' interval in between to test the repeatability of the measurement, without the intent to assess the personality of the fish. For every assay, the water was maintained at 17°C ± 1°C, and black plastic blinds surrounded the experimental setups to avoid disturbances. Fish behaviour was recorded using two cameras (Logitech C920) located 1 m above water level and the iSpy software (iSpy 64 V7.2.1.0). Data acquisition for all assays was conducted on EthovisionXT 11 software (Noldus et al. 2001).

For the risk‐taking assays, the setup included eight individual plastic tanks (28 × 18 cm) filled with 7.5 cm of thermoregulated and oxygenated water. The fish were first tested in a shelter assay with an opaque cover (15 × 8 cm) placed on the water surface on one side of the tank, used as a shelter. The fish were then tested in an open field assay by removing the shelter. At the start of each trial, one fish was introduced into each tank and left undisturbed for 5 min. The fish behaviour was then recorded for 10 min for each assay. At the end of the trial, the mass and total length of the fish were measured. The variable extracted in the shelter assay was the cumulative time spent outside the shelter (s), used as a proxy for risk‐taking. For the open field assay, the variable extracted by the software was the total distance moved by the fish (cm), used as a proxy for activity.

For the sociability assays, the setup included four experimental tanks, each divided into three sections: a central “focal” section (36 × 20.5 cm) and two side “stimulus” sections (both 10.5 × 20.5 cm), separated by a transparent plastic wall. The tanks were filled with 10 cm of water for the trials, with half of the water changed and refilled between each trial to maintain temperature and oxygen levels. At the start of each trial, groups of five conspecific fish, from the same population and same rearing environment but unfamiliar to the focal fish, were placed on either side of the “stimulus” section and left undisturbed for 3 min. The other “stimulus” section was left empty. The focal fish was then introduced in the “focal” section inside a transparent plastic cylinder for 3 min, after which the cylinder was removed, and the fish's behaviour was recorded for 15 min. At the end of the trial, the mass and total length of the focal fish were measured. For the second series of assays, the conspecifics were placed in the other “stimulus” section. The variables extracted from these assays were: fish total distance moved (cm), used as a proxy for activity in social context, and the cumulative time spent in the quarter of the “focal” section, the closest to the conspecifics (s), used as a proxy for sociability.

### 2.7 Statistical Analysis

All the statistical analyses were performed using Rstudio version 4.3.1. General linear mixed models were used to analyse the different variables. The wild population, the parental and the present environments, as well as their interactions, were used as fixed effects, and the fish family was used as a random effect for all the variables.

For the metabolic rate variables (SMR and AS) as well as EPOC and *P*_crit_, the mass of the fish at the time of the respirometry was used as a covariate and the firesting number, the respirometry chamber, the fish rearing tank, and the date of the respirometry were used as random effects. Regarding the loss of equilibrium (LOE), the mass of the fish at the time of the challenge was used as a covariate, whereas the hypoxia box number, the side of the box, the fish rearing tank, the date of the challenge, and the trial within the day were used as random effects. Concerning the growth (SGR), the initial length was additionally used as a covariate and the fish rearing tank as a random effect. For the *U*_crit_, the length of the fish at the time of the swimming was used as a covariate and the fish rearing tank, the date of swimming, and the swimming trial within the day were used as random effects. Finally, regarding the behavioural variables, the length at the time of the assays and the round were also used as covariates and the testing tanks, fish rearing tank, date of the trial, trial within the day, and fish identification tag were set as random effects. For the behavioural variables from the sociability assay, the “stimulus” position was used as a fixed effect in the model (Back or Front). For each variable, the models were reduced and selected according to their Akaike information criterion (AIC) to determine the best model. Normality and homoscedasticity of the model residuals were tested, using Shapiro's and Levene's tests, respectively. If the assumptions were not respected, a rank transformation was applied. Among all the variables tested, AS, *P*_crit_, EPOC, LOE, *U*_crit_, SGR, total distance moved in an open field and the two sociability variables were rank transformed. The final model selection can be found in Table S3. A *post hoc* Tukey's test was used to assess significant differences among the groups. The repeatability of the behaviour measurement between trials was tested following a Gaussian distribution when the homoscedasticity was respected and using a Spearman correlation if the homoscedasticity was not respected. All the individual behaviour measurements were repeatable, with the exception of the risk‐taking time spent outside the shelter being close to the repeatability threshold (Table S4). The significance threshold of *α* = 0.05 was used in all statistical tests.

## 3 Results

### 3.1 Aerobic Metabolism

**FIGURE 2:**
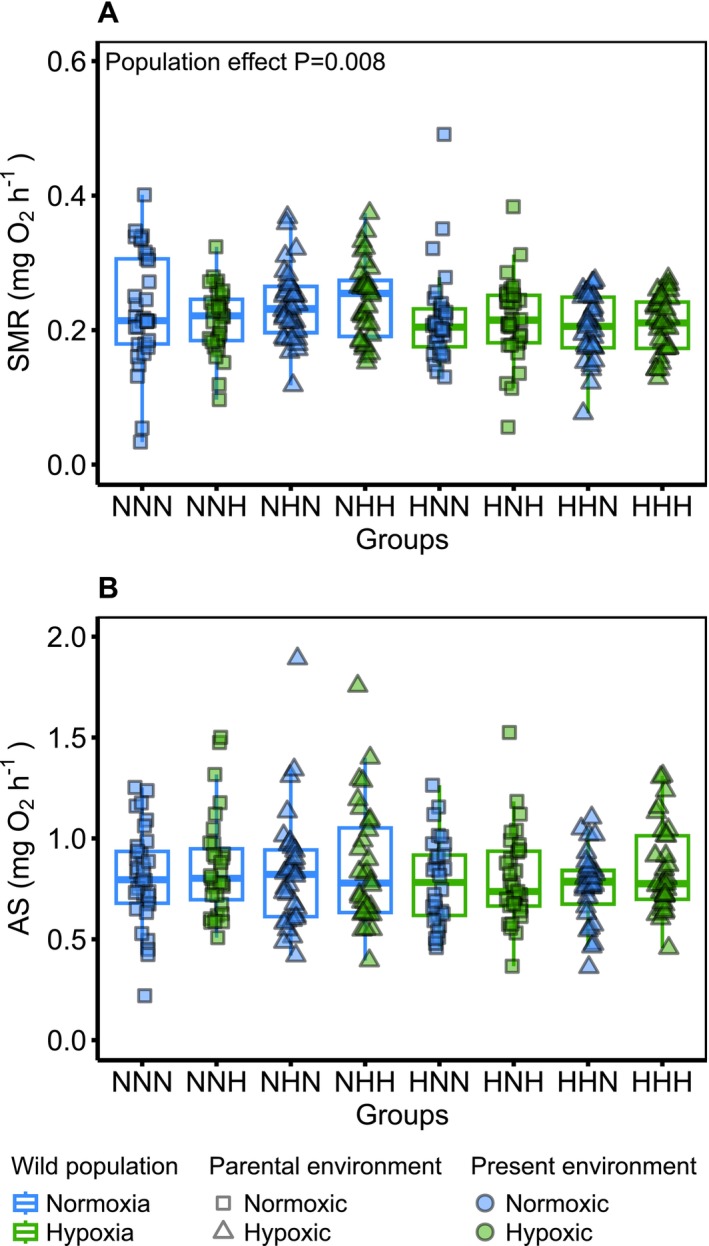
Aerobic metabolism of the different groups depending on their wild population, parental and present environments. (A) Standard metabolic rate (SMR, mg O_2_ h^−1^, adjusted to the mean mass of the fish, i.e., 0.83 g). (B) Aerobic scope (AS, mg O_2_ h^−1^, adjusted to the mean mass of the fish, i.e., 0.83 g). In total, 251 fish were used (*n* = 30–33 fish per group). The wild population is represented by the first letter of each group (N for normoxia and H for hypoxia) as well as the colour of the boxplot outline (blue for normoxia and green for hypoxia). The parental environment is indicated by the second letter of each group (N for normoxic and H for hypoxic) and the shape of the data point (square for normoxic and triangle for hypoxic). The present environment is indicated by the last letter of each group (N for normoxic and H for hypoxic) and in the colour of the data point (blue for normoxic and green for hypoxic). *p*‐value of significant fixed effect is displayed in the top left corner.

No interaction was kept after the model selection for the SMR but a non‐significant interaction between the wild population and parental environment was kept for the AS (GLM; *F*_(1,245)_ = 1.9, *p* = 0.16). The present environment had no significant effect on the SMR (GLMM; *F*_(1,13)_ = 0.45, *p* = 0.51). However, the wild population had a significant effect on the SMR (GLMM; *F*_(1,220)_ = 7.1, *p* = 0.008, Estimate = −0.013 ± 0.004) with fish from the wild hypoxia population having a lower SMR than those from the normoxia population (Figure 2A). The parental environment also had a marginal effect (GLMM; *F*_(1,215)_ = 3.3, *p* = 0.069) with the offspring from parents reared in the hypoxic environment having a marginally higher SMR than the offspring of parents reared in normoxic environments. The wild population had a marginal effect on the AS (GLM; *F*_(1,245)_ = 3.15, *p* = 0.076), with fish from the hypoxia population having the tendency for a lower AS than the fish from the normoxia population (Figure 2B). Finally, the parental and present environments had no significant effect on the AS (GLM; *F*_(1,245)_ = 0.158, *p* = 0.69; GLM; *F*_(1,245)_ = 2.1, *p* = 0.148, respectively).

### 3.2 Recovery Post‐Exercise

**FIGURE 3:**
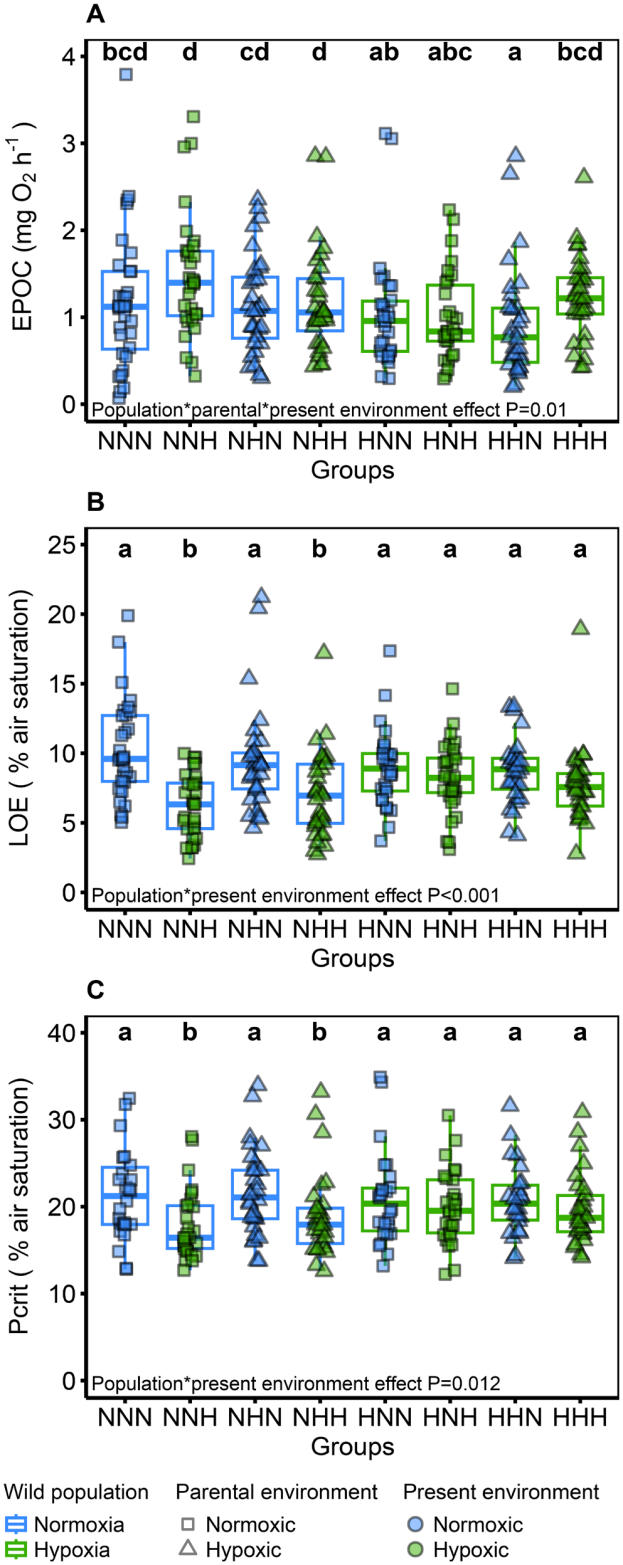
Recovery post‐exercise and hypoxia tolerance thresholds of the different groups depending on their wild populations, parental and present environments. (A) Excess post‐exercise oxygen consumption (EPOC, mg O_2_ h^−1^). In total, 249 fish were used (*n* = 30–32 fish per group). (B) Loss of equilibrium (LOE, % air saturation). In total, 251 fish were used (*n* = 30–33 fish per group) (C) Critical oxygen tension (*P*_crit_, % air saturation). In total, 224 fish were used (*n* = 26–30 fish per group). The wild population is represented by the first letter of each group (N for normoxia and H for hypoxia) as well as the colour of the boxplot outline (blue for normoxia and green for hypoxia). The parental environment is indicated by the second letter of each group (N for normoxic and H for hypoxic) and the shape of the data point (square for normoxic and triangle for hypoxic). The present environment is indicated by the last letter of each group (N for normoxic and H for hypoxic) and by the colour of the data point (blue for normoxic and green for hypoxic). Different letters indicate significant differences among the groups (GLMM: *p* < 0.05).

An interaction between the wild populations, the parental and the present environments was kept after the final model selection (GLMM; *F*_(1,221)_ = 6.73, *p* = 0.01) (Figure 3A). Fish from the wild hypoxia population, with parents exposed to a fluctuating hypoxic environment and themselves reared in a normoxic environment (HHN), were displaying a significantly lower EPOC than the fish from the wild hypoxia population with parents and themselves reared in a fluctuating hypoxic environment (HHH), and all the fish from the wild normoxia environment, in which all the groups were similar.

### 3.3 Hypoxia Tolerance Thresholds

An interaction between the wild population and the present environment was observed after the model selection for LOE in a hypoxia challenge (Figure 3B) and *P*_crit_ (Figure 3C) (GLMM; *F*_(1,226)_ = 11.6, *p* < 0.001; GLMM; *F*_(1,201)_ = 6.3, *p* = 0.012; respectively). The fish from the wild normoxia population reared in a fluctuating hypoxic environment reached their LOE and *P*_crit_ at a significantly lower oxygen percentage than the fish from the normoxia population reared in normoxic environments and the fish from the wild hypoxia population, regardless of their rearing environment. Therefore, these fish were more tolerant to hypoxia and were able to maintain their aerobic metabolism activity at a lower oxygen level than any other group. The parental environment had no significant effect on the LOE or *P*_crit_ (GLMM; *F*_(1,231)_ = 0.27, *p* = 0.59; GLMM; *F*_(1,201)_ = 0.01, *p* = 0.9; respectively).

### 3.4 Growth and Swimming Performance

**FIGURE 4:**
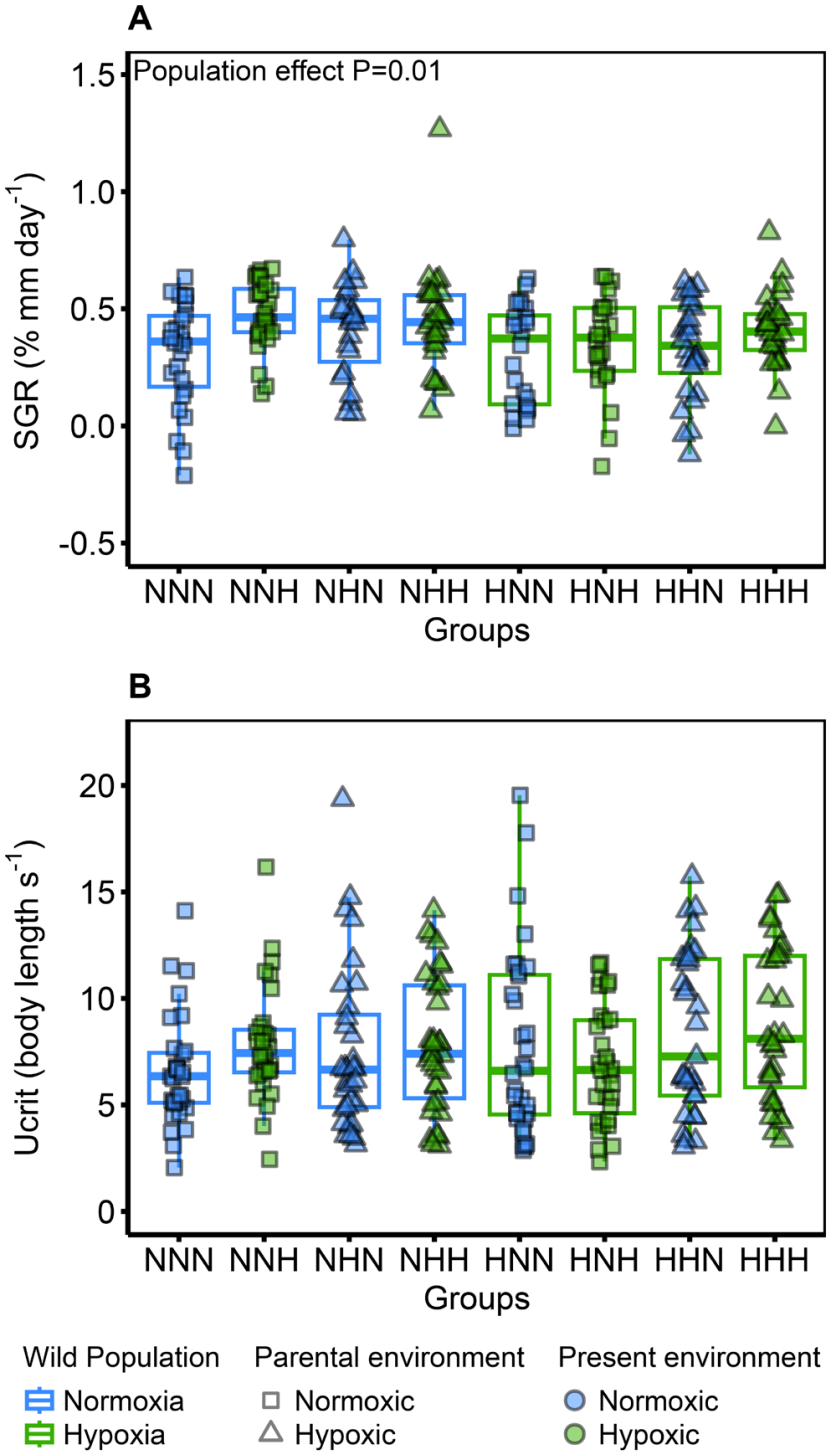
Specific growth rate and swimming performance of the different groups depending on their wild population, parental and present environments. (A) Specific growth rate (SGR, % mm day^−1^). In total, 209 fish were used (*n* = 23–28 fish per group). (B) Critical swimming speed (*U*_crit_, body length s^−1^). In total, 253 fish were used (*n* = 31–34 fish per group). The wild population is represented by the first letter of each group (N for normoxia and H for hypoxia) as well as the colour of the boxplot outline (blue for normoxia and green for hypoxia). The parental environment is indicated by the second letter of each group (N for normoxic and H for hypoxic) and the shape of the data point (square for normoxic and triangle for hypoxic). The present environment is indicated by the last letter of each group (N for normoxic and H for hypoxic) and by the colour of the data point (blue for normoxic and green for hypoxic). *p*‐value of significant fixed effects are displayed in the top left corner.

No interaction was kept after the model selection for the SGR; the only significant effect was the wild population (GLMM; *F*_(1,197)_ = 6.1, *p* = 0.01, Estimate = −0.068 ± 0.02) and a marginal effect of the present environment (GLMM; *F*_(1,18)_ = 3.7, *p* = 0.06). Fish from the wild hypoxia population had a significantly slower growth than the fish from the wild normoxia population, and fish reared in hypoxic environments tended to have a faster growth than fish reared in normoxic environments (Figure 4A). The parental environment had no effect on the SGR (GLMM; *F*_(1,194)_ = 2.6, *p* = 0.1). For the swimming performance, the wild populations (GLMM; *F*_(1,50)_ = 0.82, *p* = 0.36), the parental environment (GLMM; *F*_(1,46)_ = 1.48, *p* = 0.22), and the present environment (GLMM; *F*_(1,227)_ = 0.14, *p* = 0.7) had no effect on *U*_crit_ (Figure 4B).

### 3.5 Risk‐Taking and Social Behaviour

**FIGURE 5:**
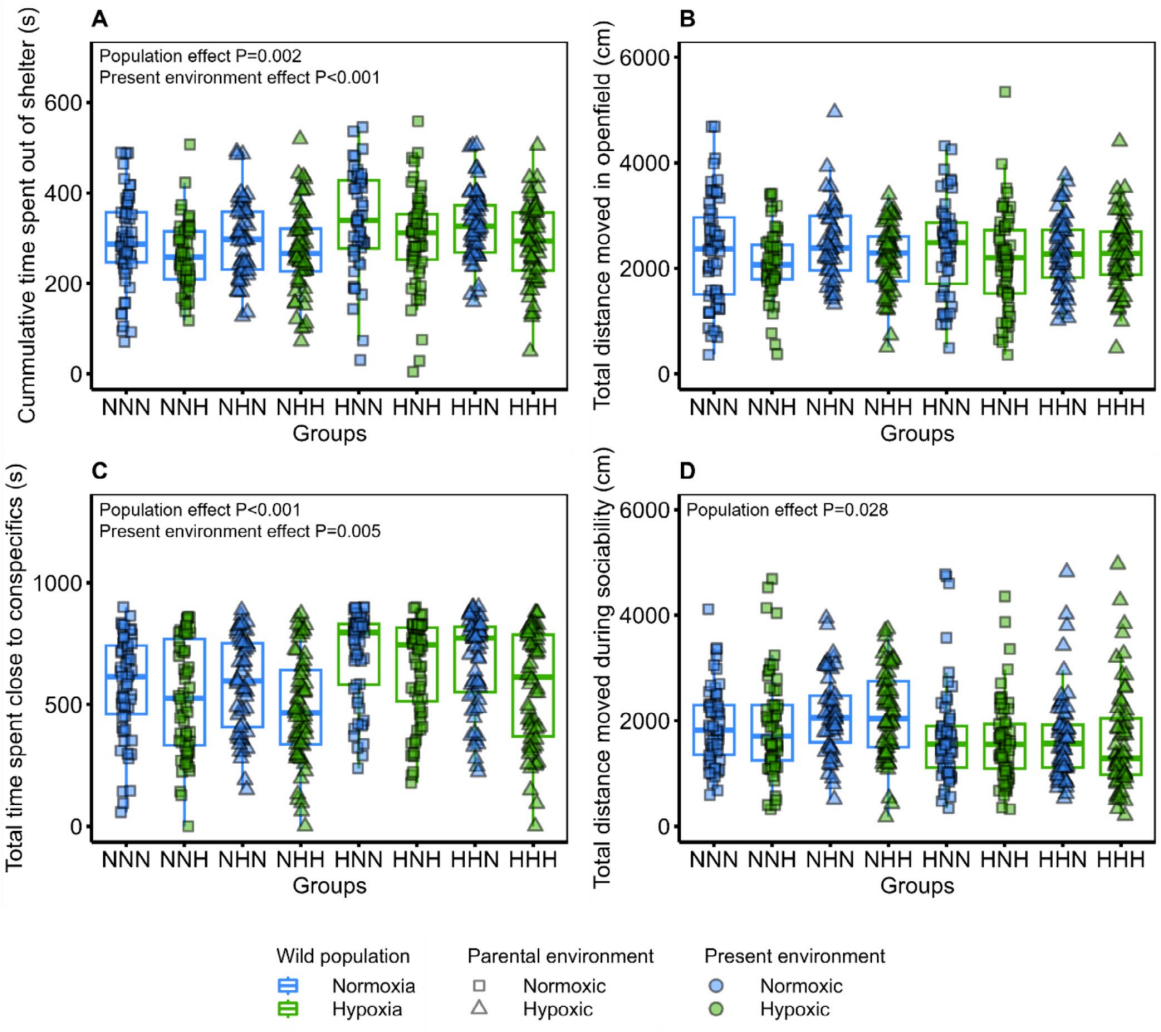
Risk‐taking and sociability behaviour of fish depending on their wild population, parental and present environment. (A) Cumulative time spent outside the shelter (seconds), (B) total distance moved in an open field (cm), (C) total time spent close to conspecifics in the sociability trial (seconds) and (D) total distance moved during the sociability trial (cm). In total, 222 fish were used for the risk‐taking assays (*n* = 25–31 fish per group) and 241 fish for the sociability assays (*n* = 27–32 fish per group). The wild population is represented by the first letter of each group (N for normoxia and H for hypoxia) as well as the colour of the boxplot outline (blue for normoxia and green for hypoxia). The parental environment is indicated by the second letter of each group (N for normoxic and H for hypoxic) and the shape of the data point (square for normoxic and triangle for hypoxic). The present environment is indicated by the last letter of each group (N for normoxic and H for hypoxic) and by the colour of the data point (blue for normoxic and green for hypoxic). P‐values for significant fixed effects are displayed in the top left corner of each figure.

Regarding the risk‐taking of the fish, no interaction was maintained after the model selection for the time the fish spent outside a shelter (Figure 5A) or the total distance moved in an open field (Figure 5B). However, the present environment had a significant effect on the time spent outside the shelter and a marginal effect on the total distance moved in an open field. (GLMM; *F*_(1,214)_ = 18.8, *p* < 0.001, Estimate = −48.2 ± 8.81, GLMM; *F*_(1,14)_ = 4.48, *p* = 0.053, for the time spent outside the shelter and the total distance moved respectively). The fish reared in a fluctuating hypoxic environment spent significantly less time outside the shelter and tended to cover less distance than fish reared in a normoxic environment. The fish reared in a fluctuating hypoxic environment were taking less risk and had the tendency to be less active than the fish reared in a normoxic environment. The wild population also had a significant effect on the time spent outside the shelter (GLMM; *F*_(1,218)_ = 9.55, *p* = 0.002, Estimate = 27.7 ± 8.9). The fish from the wild hypoxia population spent a longer time outside the shelter, so they were taking more risks than the fish from the wild normoxia population. The parental environment had no significant effect on the time spent outside the shelter (GLMM; *F*_(1,212)_ = 0.02, *p* = 0.88) and the wild population and parental environment had no significant effect on the total distance moved (GLMM; *F*_(1,215)_ = 0.79, *p* = 0.373; GLMM; *F*_(1,208)_ = 0.58, *p* = 0.44, respectively).

Regarding the sociability of the fish, a marginal interaction between the parental environment and the present environment was maintained after the model selection on the time the fish spent close to their conspecifics (GLMM; *F*_(1,201)_ = 2.7, *p* = 0.09). Fish from parents reared in fluctuating hypoxic environments and reared in the same environment tended to spend less time close to their conspecifics than fish from parents reared in either environment but reared in normoxic environments. However, the present environment had a significant effect (GLMM; *F*_(1,13)_ = 10.9, *p* = 0.005, Estimate = −50 ± 14.4), with fish reared in a fluctuating hypoxic environment spending significantly less time close to their conspecifics and were thus less social than the fish reared in normoxic environments (Figure 5C). In addition, the wild population environment had a significant effect (GLMM; *F*_(1,40)_ = 23.2, *p* < 0.001, Estimate = 69.6 ± 14.5). The fish from the wild hypoxia population spent significantly more time close to their conspecifics and were more social compared to the fish from the wild normoxia population. For the total distance moved during the sociability trials (Figure 5D), an interaction was kept between the wild population and parental environment after the model selection (GLMM; *F*_(1,228)_ = 4.8, *p* = 0.028). However, after the *post hoc* analysis, only the wild population environment had a significant effect on the total distance moved, with fish from the hypoxia population covering significantly less distance and being less active than the fish from the normoxia population. The present environment had no significant effect on the total distance moved during the sociability trial (GLMM; *F*_(1,236)_ = 1.71, *p* = 0.191).

## 4 Discussion

The goal of this study was to highlight the potential phenotypic responses that three‐spined sticklebacks could have within and across generations when facing hypoxia. When exposed to a fluctuating hypoxic environment within a generation, fish decreased their risk‐taking and sociability behaviour as we expected. However, interestingly, fish from the hypoxia population were more prone to take risks and were more social. This highlights that the plastic responses did not seem to drive the adaptation of these same behavioural traits, which were instead going in the opposite direction. Furthermore, the hypoxia population also had a lower SMR and growth, indicating that fish may have experienced adaptation for these traits. We also found that the fish from the hypoxia population showed signs of adaptation in their plasticity, losing their direct plasticity capacity for hypoxia tolerance. Indeed, the hypoxia population had similar LOE and *P*_crit_ regardless of the environment, whereas the fish from the normoxia population displayed a higher hypoxia tolerance, reaching lower LOE and *P*_crit_ when exposed to a fluctuating hypoxic environment in the present generation. However, the hypoxia population displayed plasticity for their recovery post‐exercise (EPOC), with a faster recovery in fish from parents reared in a fluctuating hypoxic environment and themselves reared in a normoxic environment compared to when the fish were reared in a fluctuating hypoxic environment, whereas fish from the normoxia population were all similar, additionally supporting some adaptation in direct plasticity. Finally, we didn't observe intergenerational plasticity for the trait measured. Overall, our results suggested that fish might be resilient to within and across generations exposure to hypoxia, with plastic responses potentially giving time for the genetic adaptation to take place, adaptation which seemed to have occurred even in a short climate change timeframe.

An overall reduction of the risk‐taking, activity and sociability was observed in the fish exposed to fluctuating hypoxia in their present environment, as we anticipated, hinting at phenotypic plasticity. As the fish were exposed to hypoxia only during the night, and the behavioural differences were observed during the day, once the fish were returned to normoxia, it seems that some carry‐over effects of the night hypoxia still impacted the behaviour of the fish during the day. It is possible that the fish were increasing their anaerobic metabolism during the night to compensate for the limited amount of oxygen available to produce energy (Borowiec et al. 2015; Sneddon and Yerbury 2004), potentially increasing the production of metabolites such as lactate. The night hypoxia might thus have created an oxygen debt that would need to be paid when the fish returned to normoxia the following day (Borowiec et al. 2015, 2018; Sneddon and Yerbury 2004). Similarly, previous studies showed lasting impacts of hypoxia on the fish nervous system and behaviour in the recovery period (Braga et al. 2013; Sánchez‐García et al. 2019). However, we did not observe any plasticity of SMR and AS during the day, contrary to our hypothesis, suggesting that the fluctuating hypoxia did not induce long‐lasting costs in the oxygen needed to fuel the aerobic metabolism during the day. The fish might still likely reduce their risk‐taking and activity during the night hypoxia to conserve energy (Sneddon and Yerbury 2004; Kramer 1987; van Raaij et al. 1996), while reducing their time close to their conspecifics to maximise their individual oxygen uptakes and avoid exacerbating hypoxia, which can occur among large amounts of fish schooling (Domenici et al. 2017). The fish seem to have carried over this behavioural strategy even during the day once back in normoxia, highlighting that in a fluctuating environment, fish might retain their plastic responses, even when returning to their initial environment. Overall, these results showed that fish were able to cope with within‐generation hypoxia through their plastic behaviour responses, highlighting their resilience in rapid and fluctuating environmental changes.

When fish were exposed to hypoxia across generations, the behavioural responses were, in general, going in the opposite direction to the within‐generation responses, with fish of the hypoxia population showing higher risk‐taking and sociability than fish from the normoxia population. We observed these results after two consecutive generations of fish reared in a common garden, suggesting potential adaptation to the wild hypoxic environments. The wild environment may have created some selection pressure, even in the short climate change time scale, increasing the fitness of fish taking more risks and being more social. Fish that took more risks could have been able to explore more of their environment, potentially increasing their chance of finding food (dos Santos et al. 2023; King et al. 2013) but also of finding areas less impacted by hypoxic events (Pihl et al. 1991). Even though increased risk‐taking could have made fish more vulnerable to predation, the hypoxic environment still appeared to favour those that took more risks. The benefits of finding food or more suitable habitats likely outweighed the costs of predation (Hulthén et al. 2017). Being more social and living in the proximity of conspecifics could have also helped in the early detection of predators, predator confusion, and more efficient foraging (Domenici et al. 2017). In addition, fish living in shoals could be more efficient at finding areas less impacted by hypoxic events by increasing the search effort compared to individual fish (Domenici et al. 2017; Killen et al. 2017). Similarly, to the risk‐taking, it seemed that the cost of being social and close to conspecifics, potentially exacerbating hypoxia, was outweighed by the benefits of being in shoals for the hypoxia population. We cannot exclude that other environmental parameters such as water temperature, water flow, or interspecies variation could have also contributed toward the behavioural adaptation of the fish across generations. Indeed, our hypoxia population also experienced higher summer temperature. However, a previous study showed that stickleback populations adapted to warm environments were actually less social (Pilakouta et al. 2023). Therefore, it is highly probable that the main differences between our two populations were due to the occurrence of strong hypoxia events in the wild environments. Investigating additional populations strongly exposed to hypoxia would be interesting to provide further support to our findings.

These potential adaptations of fish behaviour were also in the opposite direction to the within‐generation behavioural responses. This pattern of opposition between plasticity and adaptation was previously described and adaptation would generally rather reverse than reinforce plasticity (Ho and Zhang 2018). When facing environmental changes, organisms' fitness would usually be reduced despite the plastic responses and would then be recovered through subsequent adaptation (Ho and Zhang 2018). In our study, although the fish first showed plasticity, reducing their risk‐taking and social behaviour during their recovery, the wild hypoxic environment may have favoured a selection for a faster recovery post hypoxia, allowing them to show increased risk‐taking and social behaviour. Although the emergency responses through plasticity would be necessary for the fish to survive to their immediate new environmental condition, it will not bring the organisms closer to a new optimum and rather would give time for the genetic adaptation to occur and increase the fitness of fish across generations.

A potential adaptation of the aerobic metabolism was also observed, with a reduction of SMR, as expected, and a slower fish growth in the hypoxia population, but no adaptation of the AS. Similar adaptations in aerobic metabolism and growth were also previously described in fish species living in chronically hypoxic environments (Mandic and Regan 2018; Joyner‐Matos and Chapman 2013; Wang et al. 2009). The wild environment of the fish from the hypoxia population seems to have induced selection, favouring a lower overall energy demand and usage. Fish with lower SMR would require less oxygen to sustain their anabolism and catabolism activity and maintain their homeostasis (Chabot et al. 2016; Metcalfe et al. 2016), which could contribute to more efficient usage and conservation of energy in their wild hypoxic environment (Mandic and Regan 2018; Wang et al. 2009; Mandic et al. 2009; Reardon and Chapman 2009, 2012). Previous studies on fish adapted to high altitude hypoxia, in the Tibetan plateau, also showed a positive selection of genes associated with a reduction of oxygen consumption and metabolic energy expenditure (Yang et al. 2021), hinting at a possible similar adaptation process over millions of years. In addition, a low oxygen environment might have favoured selection for slower growth as faster growing fish, having higher energy demands, could be unable to get the oxygen needed to sustain their energy requirements (Joyner‐Matos and Chapman 2013; Reardon and Chapman 2012; Le Roy et al. 2017). Therefore, part of the energy saved with a lower SMR and slower growth could be allocated toward keeping high risk‐taking activity observed in the hypoxia population. Overall, it seems that some fish might be able to adapt to hypoxia in a climate change time scale by adjusting their aerobic components such as decreasing their basal metabolic energy demand and their growth rate.

We also observed signs of adaptation of the fish plasticity capacity, especially on traits related to hypoxia tolerance threshold. The fish from the normoxia population exposed to fluctuating hypoxia in their present environment were having a higher overall hypoxia tolerance with a lower LOE and lower oxygen limits for maintaining their basal metabolism (*P*_crit_), as we expected. Furthermore, in contradiction with our initial assumptions that fish from the hypoxia population would show a stronger plastic response, the fish from the hypoxia population showed no difference in their hypoxia tolerance across rearing environments, with a hypoxia tolerance similar to the one observed in the fish from the normoxia population never exposed to hypoxia, hinting at a possible adaptation toward a loss of plasticity for these traits. In addition, the fish from the hypoxia population with parents reared under hypoxic environment and themselves reared under normoxic environment had a faster recovery post‐exercise (EPOC) compared to the fish reared under hypoxic environment, highlighting a potential adaptation of the combination of intergenerational and direct plasticity. As environments with high variation generally favour plasticity (Burton et al. 2022; Gomez‐Mestre and Jovani 2013; Masel et al. 2007), we were expecting that the wild fluctuating hypoxic environment would have favoured a selection toward high plasticity for hypoxia tolerance. However, our results hint at a loss of plasticity instead, especially in LOE and *P*_crit_. One possible explanation could be that although hypoxic events occurred frequently in the wild environment of the hypoxia population, maintaining plasticity in hypoxia tolerance threshold, that partly rely on anaerobic metabolism, could have come at a cost for the fish's fitness (Murren et al. 2015; Burton et al. 2022). In addition, such costs could have been redundant with the adaptation to hypoxia in aerobic metabolism, as the fish could potentially rely on their adaptation in aerobic metabolism to cope with the oxygen reduction in their environment. Their need to rely on plasticity in traits related to their anaerobic metabolism would become limited, ultimately leading to this plasticity loss. However, although the plasticity of some anaerobic related traits seems to be lost through adaptation, other traits seem to be selected toward an increase of their plasticity, such as the EPOC in hypoxic parental environments. A lower EPOC, indicating a reduction of the oxygen needed to eliminate the lactic acid build‐up and a faster replenishment of the cellular oxygen and ATP stores after activity (Pang et al. 2020), could improve fish performance and fitness. Overall, these results highlighted that in addition to adaptation in their aerobic metabolism, fish might adapt their hypoxia tolerance threshold and anaerobic metabolism plasticity. Therefore, such adaptation of plasticity might deeply impact the organisms' capacity to cope with future fluctuations in their environment.

Surprisingly, no intergenerational plasticity to fluctuating hypoxia was observed for all the traits measured in contrast to our hypothesis. Previous studies highlighted that short parental exposure to constant hypoxia increased the offspring's body size and hypoxia tolerance during the larval stage (Ho and Burggren 2012; Ragsdale et al. 2022). The lack of intergenerational plasticity in our results suggests that hypoxia, either fluctuating or of longer exposure, might not induce similar intergenerational plasticity responses in adult fish. However, because the measurements were performed on adult fish, we cannot exclude that the fish displayed any intergenerational plasticity responses throughout their lifecycle. Intergenerational plasticity might have occurred at the larval stage, enhancing larvae survival, but could not necessarily have been kept at later life stages, when individuals are generally less sensitive to their environments (Bautista and Crespel 2021), or it could also have been impacting other traits not measured here. Although the parental environment didn't influence the offspring phenotype, we cannot rule out that the grandparental environment could have an effect through trans‐generational plasticity. Plasticity of a trait could be shaped by the grandparents' environment, showing delayed response to the next generations, or a combination of both parental and grandparental environments could be required to induce significant cross‐generational phenotypic responses (Bell and Hellmann 2019; Le Roy et al. 2017). It is also possible that these traits are not impacted by cross‐generational plasticity to fluctuating hypoxia but only by within generation plasticity or adaptation processes. In such case, cross‐generational plasticity wouldn't seem to be facilitating evolution. This overall absence of intergenerational plasticity could have repercussions on the population resilience when facing climate change induced hypoxic events. If fish population lack the ability to buffer the effects of the environment with cross‐generational plasticity until genetic assimilation can take place, they might have to rely only on within generation plasticity to cope with the environmental change before adaptation occurs.

With this study, we brought new light onto the possible different responses expressed by fish when exposed to fluctuating hypoxia within and across generations. Fish were able to modulate their behaviour in response to fluctuating hypoxia through direct plasticity, which could in turn give more time for adaptation to occur. Fish were able to show signs of adaptation to hypoxia in the short timeframe of climate change, with behavioural adaptation going in the opposite direction of the plastic responses but also adaptation on the basal aerobic metabolism and growth, as well as adaptation on the plasticity of hypoxia tolerance thresholds, leading to either a loss or a maintenance of the plasticity for these traits. A more in‐depth analysis of the genomic differences between the wild populations would still be needed to confirm the potential adaptation highlighted by our findings. However, the fish did not seem to display intergenerational plasticity, potentially affecting their ability to buffer the effect of fluctuating hypoxia across generations, whereas adaptation takes place. Overall, our study reveals that fish have the capacity to deal with climate change hypoxic events through within‐generation plasticity, buffering environmental changes until adaptation can occur. In the future, it will also become important to integrate fluctuating environmental parameters, such as hypoxia, with other environmental parameters to more faithfully represent the complexity found in the wild. It is only by investigating more accurate representations of future wild environments that we can deepen our understanding of how freshwater fish could cope with the upcoming environmental change across time.

## Author Contributions

**Ludovic Toisoul:** data curation (equal), formal analysis (lead), investigation (lead), visualisation (lead), writing – original draft (lead). **Alycia Valvandrin:** data curation (equal), investigation (equal), writing – review and editing (equal). **Luisa Bermejo Albacete:** investigation (supporting), resources (lead). **Katja Anttila:** supervision (supporting), writing – review and editing (equal). **Amélie Crespel:** conceptualization (lead), funding acquisition (lead), methodology (lead), project administration (lead), resources (equal), supervision (lead), validation (lead), writing – review and editing (lead).

## Funding

This work was supported by Research Council of Finland, 343562. Koneen Säätiö, 201907804.

## Conflicts of Interest

The authors declare no conflicts of interest.

## Supporting information


**Table S1:** Environmental parameters of the wild population streams before and at the time of the sampling.
**Figure S1:** Wild population habitats and water oxygen profiles. (A) Picture of the habitat of the two wild populations at the time of the 2020 sampling, left Lottbek and right Ottersbek. (B) Ottersbek water oxygen concentration profile (mg L^−1^) over a period of 164 days between May and October 2012.
**Table S2:** Number of fish used for each group for each of the phenotypic traits. The first letter of the group, either normoxia (N) or hypoxia (H), represents the wild population. The second and third letters represent respectively the parental and present environment.
**Table S3:** Final model selected for each of the phenotypic traits. Random effects are represented between brackets. Ranked indicate if the trait was ranked transformed during the analysis.
**Table S4:** Repeatability of the risk‐taking and social behaviour variables.

## Data Availability

All data that support the findings of the study are openly available in Figshare at 10.6084/m9.figshare.30265300.
