# Supplementary material for: Resilience in a Hypoxic World: Fish Respond Through Plasticity in Their Behaviour, Whereas Adaptation and Adaptation of Plasticity in the Behaviour and Metabolism Occur
Source: Ecol Evol. 2026 Feb 24;16(2):e73128. doi: 10.1002/ece3.73128 (PMC12930215; doi:10.1002/ece3.73128)
Supplement: Supplementary file 1 — Table S1: Environmental parameters of the wild population streams before and at the time of the sampling. Figure S1: Wild population habitats and water oxygen profiles. (A) Picture of the habitat of the two wild populations at the time of the 2020 sampling, left Lottbek and right Ottersbek. (B) Ottersbek water oxygen concentration profile (mg L−1) over a period of 164 days between May and October 2012. Table S2: Number of fish used for each group for each of the phenotypic traits. The first letter of the group, either normoxia (N) or hypoxia (H), represents the wild population. The second and third letters represent respectively the parental and present environment. Table S3: Final model selected for each of the phenotypic traits. Random effects are represented between brackets. Ranked indicate if the trait was ranked transformed during the analysis. Table S4: Repeatability of the risk‐taking and social behaviour variables. [file ECE3-16-e73128-s001.docx]

**Resilience in a hypoxic world: fish respond through plasticity in their behaviour, while adaptation and adaptation of plasticity in the behaviour and metabolism occur**

Authors: Ludovic Toisoul^1*^, Alycia Valvandrin^1^, Luisa Bermejo Albacete^1^, Katja Anttila^1^, Amélie Crespel^1^

**Table S1:** **Environmental parameters of the wild population streams before and at the time of the sampling.**

|  | **Lottbek year 2011** | | | **Lottbek 2020** | **Ottersbek Summer 2012** | | | **Ottersbek 2020** |
| --- | --- | --- | --- | --- | --- | --- | --- | --- |
|  | Average | Min | Max | September Sampling | Average | Min | Max | September Sampling |
| **Temperature (°C)** | 9.1 | 0.5 | 17.1 | 15.2 | 16.8 | 12 | 21.7 | 17.7 |
| **Oxygen (mg L^-1^)** | 9.9 | 6.7 | 12.9 | 6.3 | 5.7 | 1.1 | 11.5 | 1.8 |
| **pH** | 7.4 | 6.9 | 7.8 | 6.9 | 7.3 | 7.1 | 7.4 | 7.2 |


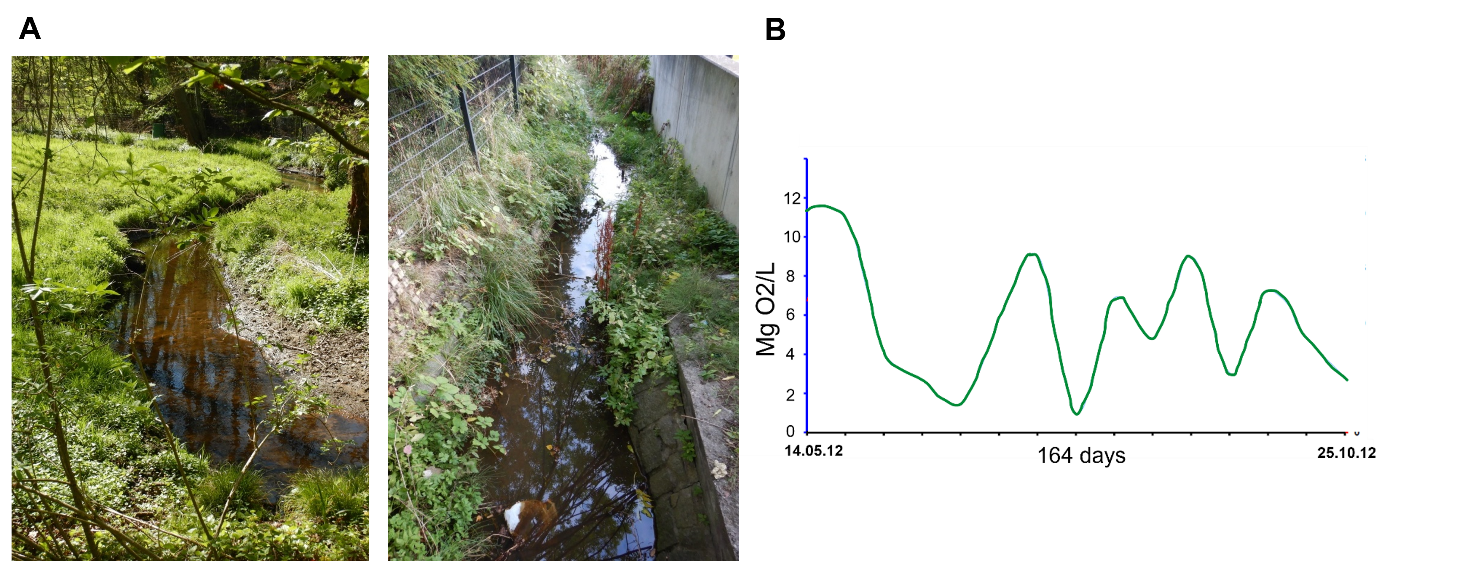


**Figure S1**: **Wild population habitats and water oxygen profiles**. A) Picture of the habitat of the two wild populations at the time of the 2020 sampling, left Lottbek and right Ottersbek B) Ottersbek water oxygen concentration profile (mg L^-1^) over a period of 164 days between May and October 2012.

Table S2: **Number of fish used for each group for each of the phenotypic traits.** The first letter of the group, either normoxia (N) or hypoxia (H), represents the wild population. The second and third letters represent respectively the parental and present environment.

|  | | **Number of fish used for each group** | | | | | | | |
| --- | --- | --- | --- | --- | --- | --- | --- | --- | --- |
| **Phenotypic trait** | | NNN | NNH | NHN | NHH | HNN | HNH | HHN | HHH |
| Aerobic metabolism | SMR | 33 | 31 | 32 | 32 | 30 | 31 | 31 | 31 |
|  | AS | 33 | 31 | 32 | 32 | 30 | 31 | 31 | 31 |
| Anaerobic capacity | LOE | 32 | 31 | 31 | 33 | 30 | 32 | 31 | 31 |
|  | *P*_crit_ | 26 | 29 | 30 | 29 | 26 | 29 | 26 | 29 |
|  | EPOC | 32 | 31 | 32 | 32 | 30 | 30 | 31 | 31 |
| Growth and swimming performance | SGR | 26 | 28 | 23 | 28 | 24 | 24 | 28 | 28 |
|  | *U*_crit_ | 31 | 31 | 32 | 34 | 32 | 31 | 31 | 31 |
| Risk-taking behaviour | Time out shelter | 28 | 26 | 25 | 30 | 26 | 29 | 30 | 28 |
|  | Open field total distance moved | 28 | 26 | 25 | 30 | 26 | 29 | 30 | 28 |
| Social behaviour | Time close conspecific | 29 | 30 | 27 | 32 | 29 | 32 | 31 | 31 |
|  | Total distance moved | 29 | 30 | 27 | 32 | 29 | 32 | 31 | 31 |

Table S3: **Final model selected for each of the phenotypic traits.** Random effects are represented between brackets. Ranked indicate if the trait was ranked transformed during the analysis.

| **Phenotypic trait** | | **Ranked** | **Final model** |
| --- | --- | --- | --- |
| Aerobic metabolism | SMR | no | Wild + Parental + Present + Mass + (Firesting) + (Chamber) + (Tank) + (Date) |
|  | AS | yes | Wild * Parental + Present + Mass |
| Anaerobic capacity | LOE | yes | Wild * Present + Parental + Mass + (Tank) + (Hypoxia Box) |
|  | *P*_crit_ | yes | Wild * Present + Parental + Mass + (Firesting) + (Date) |
|  | EPOC | yes | Wild * Parental * Present + Mass + (Tank) + (Date) |
| Growth and swimming performance | SGR | no | Wild + Parental + Present + Length + (Tank) |
|  | *U*_crit_ | yes | Wild + Parental + Present + Length + (Swimming Trial) + (Date) + (Family) |
| Risk-taking behaviour | Time out shelter | no | Wild + Parental + Present + Length + (Testing tanks) + (Date) + (FishID) |
|  | Open field total distance moved | yes | Wild + Parental + Present + Length + Round + (Tank) + (Date) + (FishID) |
| Social behaviour | Time close conspecific | yes | Wild + Parental * Present + Stimulus position + Round + (Testing tanks) + (Tank) + (Run) + (Fam) + (FishID) |
|  | Total distance moved | yes | Wild * Parental + Present + Length + Round + (Testing tanks) + (Date) + (FishID) |

Table S4: **Repeatability of the risk taking and social behaviour variables.**

| **Phenotypic trait** | | **Statistic test** | **Repeatability (rho)** | **Pvalues** |
| --- | --- | --- | --- | --- |
| Risk-taking behaviour | Time out shelter | rpt | 0,064 | P = 0,156 |
|  | Open field total distance moved | Spearman | 0,384 | **P < 0,001** |
| Social behaviour | Time close conspecific | Spearman | 0,264 | **P < 0,001** |
|  | Total distance moved | Spearman | 0,353 | **P < 0,001** |
